# Wastewater Surveillance Can Function as an Early Warning System for COVID-19 in Low-Incidence Settings

**DOI:** 10.3390/tropicalmed8040211

**Published:** 2023-03-31

**Authors:** Mohamad Assoum, Colleen L. Lau, Phong K. Thai, Warish Ahmed, Jochen F. Mueller, Kevin V. Thomas, Phil Min Choi, Greg Jackson, Linda A. Selvey

**Affiliations:** 1 School of Public Health, Faculty of Medicine, The University of Queensland, Brisbane, QLD 4006, Australia; 2 Queensland Alliance for Environmental Health Sciences (QAEHS), The University of Queensland, Woolloongabba, QLD 4102, Australia; 3 CSIRO Land and Water, Ecosciences Precinct, 41 Boggo Road, Dutton Park, QLD 4102, Australia; 4 Health Protection Branch, Queensland Health, Brisbane, QLD 4006, Australia

**Keywords:** wastewater surveillance, SARS-CoV-2, early warning, surveillance

## Abstract

Introduction: During the first two years of the COVID-19 pandemic, Australia implemented a series of international and interstate border restrictions. The state of Queensland experienced limited COVID-19 transmission and relied on lockdowns to stem any emerging COVID-19 outbreaks. However, early detection of new outbreaks was difficult. In this paper, we describe the wastewater surveillance program for SARS-CoV-2 in Queensland, Australia, and report two case studies in which we aimed to assess the potential for this program to provide early warning of new community transmission of COVID-19. Both case studies involved clusters of localised transmission, one originating in a Brisbane suburb (Brisbane Inner West) in July–August 2021, and the other originating in Cairns, North Queensland in February–March 2021. Materials and Methods: Publicly available COVID-19 case data derived from the notifiable conditions (NoCs) registry from the Queensland Health data portal were cleaned and merged spatially with the wastewater surveillance data using statistical area 2 (SA2) codes. The positive predictive value and negative predictive value of wastewater detection for predicting the presence of COVID-19 reported cases were calculated for the two case study sites. Results: Early warnings for local transmission of SARS-CoV-2 through wastewater surveillance were noted in both the Brisbane Inner West cluster and the Cairns cluster. The positive predictive value of wastewater detection for the presence of notified cases of COVID-19 in Brisbane Inner West and Cairns were 71.4% and 50%, respectively. The negative predictive value for Brisbane Inner West and Cairns were 94.7% and 100%, respectively. Conclusions: Our findings highlight the utility of wastewater surveillance as an early warning tool in low COVID-19 transmission settings.

## 1. Introduction

From the emergence of SARS-CoV-2 in late 2019, the COVID-19 pandemic rapidly spread throughout the globe. By 1 September 2022, an estimated 608 million people had been infected, with an estimated 6.5 million fatalities [1]. People with COVID-19 may be highly infectious while showing no or minimal symptoms [2], making surveillance and control of the infection difficult to achieve through measures such as case identification and quarantining of contacts.

The shedding of viral particles via the gastrointestinal tract can occur prior to the onset of symptoms, and viral RNA can be detectable in the faecal matter of asymptomatic people [3]. Viral fragments secreted via oral, nasal, or other routes can also enter wastewater when washed off hands and bodies. Detection of viral RNA in wastewater using PCR testing can, therefore, provide opportunities for early warning of undetected SARS-CoV-2 infections in communities, particularly in low- or zero-transmission settings. When used in conjunction with epidemiological case data, wastewater detections can be useful in informing public health responses [4,5,6,7]. For example, wastewater sampling has been used to monitor and inform the public health response to COVID-19 on university campuses [4]. Wastewater sampling has also been used to identify potential mobile testing sites where there are few human cases identified but high levels of SARS-CoV-2 detected in the wastewater [5]. Wastewater sampling and testing for polioviruses led to the identification of the circulation of wild poliovirus type 1 in Israel, where there were no clinical cases. As a result, further vaccination campaigns were undertaken to prevent outbreaks of clinical cases [7]. In the Netherlands, wastewater detection of SARS-CoV-2 preceded case detection in the early stages of the pandemic [8]. Until December 2021, due to international and domestic border restrictions and the imposition of lockdowns, there was limited transmission of SARS-CoV-2 in the state of Queensland, Australia [1,9]. At that time, imported cases were confined in quarantine hotels and there were occasional clusters of limited transmission. By 1 December 2021, only 2130 cases and seven deaths had been reported in the population of ~5.2 million.

In this paper, we describe the wastewater surveillance program for SARS-CoV-2 in Queensland, Australia. We report two case studies in which we aimed to assess the potential for this program to provide early warning of new community transmission of COVID-19. Both case studies involved clusters of localised transmission, one originating in a Brisbane suburb (Brisbane Inner West cluster) in July–August 2021, and the other originating in the Far North Queensland city of Cairns, in February–March 2021.

## 2. Materials and Methods

In Queensland, a SARS-CoV-2 wastewater surveillance system commenced in July 2020 under the leadership of Queensland Health. Raw wastewater samples were collected from the inlet of wastewater treatment plants (WWTP) across Queensland by local utilities. Samples were chilled, transported to the laboratory in Brisbane, and analysed in triplicate by reverse transcriptase quantitative PCR (RT-qPCR) using a Bio-Rad CFX96 thermal cycler (Bio-Rad Laboratories, Hercules, CA, USA) [10,11,12]. More details on the operational and laboratory aspects of the program have been published previously [10,11,12].

**Figure 1:**
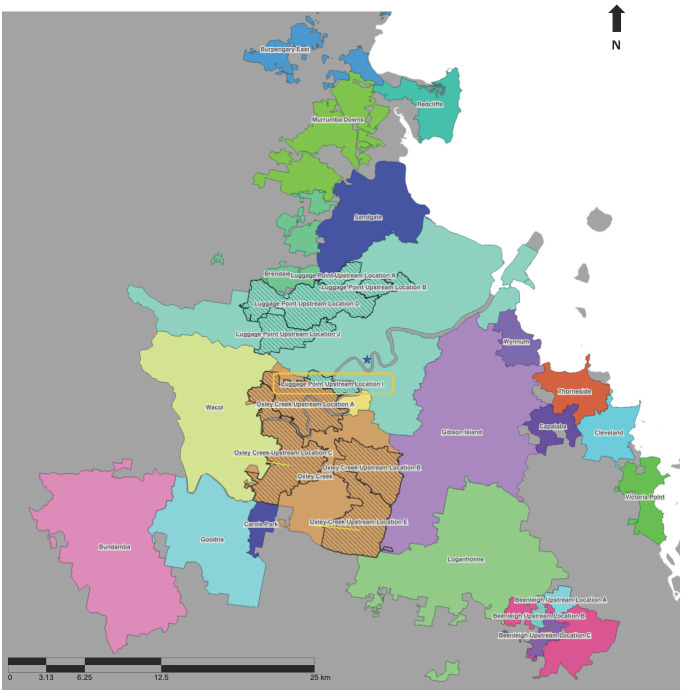
Catchment boundaries of sewage treatment plants (STPs, solid colours) and their respective upstream sampling sites (quilted pattern) across the Brisbane area. The yellow box highlights the Luggage Point upstream location I, and the star represents the Brisbane central business district. The coloured dots represent the suburbs of St Lucia (orange), Indooroopilly (blue), and Taringa (green).

As of August 2021, wastewater samples were typically collected using autosamplers as 24 h composite samples at WWTPs across 63 sites in Queensland. Some WWTPs in the greater Brisbane area are large, with the largest, Luggage Point, servicing over 800,000 residents. Due to the risk of dilution, eleven upstream sample catchment areas were selected for passive sampling [13,14] in addition to the WWTP sampling, each servicing a population of between 10,000 and 100,000 (Figure 1). Upstream sampling was undertaken through 3 or 4 day deployment of Torpedo-style passive samplers at 15 upstream locations within the Luggage Point, Oxley Creek, and the Gibson Island WWTP catchments (Figure 1) [14,15]. The minimum sampling frequency was one sample per week. Wherever feasible, samples were collected twice weekly. During periods of heightened COVID-19 transmission risk in an area, the frequency of upstream sampling in the area and surrounds increased. During the period of the two case studies, the data were reported as detected/not detected, and no RNA quantification was performed.

COVID-19 is a notifiable condition under the Queensland Public Health Act 2005 [16]. At the time of this study, Queensland cases that were not identified in hotel quarantine or were not identified as contacts of existing cases were investigated and their contacts were traced. Public health interventions of unlinked cases sometimes included a snap lockdown for a few days and/or increased testing. The two case studies were identified by the public health units through this process [15]. For the two case studies, SARS-CoV-2 wastewater surveillance data from the relevant WWTPs were merged with human COVID-19 case data. Publicly available COVID-19 case data derived from the notifiable conditions (NoCs) registry from the Queensland Health data portal [17] were cleaned and merged spatially with the wastewater data using statistical area 2 (SA2) codes [18]. Data were cleaned using JMP Pro 16 [19].

The positive predictive values (PPVs) and negative predictive values (NPVs) of wastewater detection for predicting the presence of COVID-19 reported cases were calculated for the two case study sites. PPVs were calculated as the proportion of positive wastewater detections that were associated with positive human case detections. For this calculation, reported cases of COVID-19 within a 10 day window of a wastewater detection (5 days before and 5 days after wastewater detection) were matched with a positive wastewater detection if they occurred. NPV were calculated as the proportion of negative detections that were considered true negatives (where there were no reported cases of COVID-19 within a 10 day window (5 days before and 5 days after a wastewater sample was taken).

**Figure 2:**
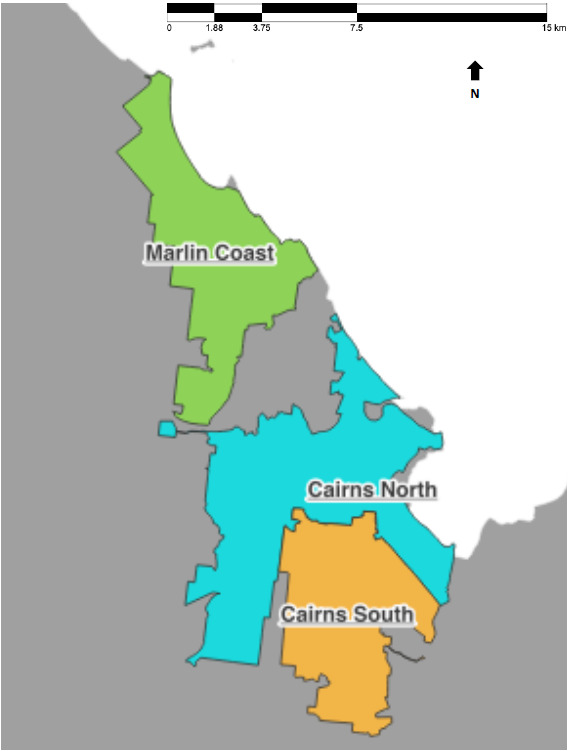
Catchment boundaries of wastewater treatment plants in the Cairns region.

The following parameters were used for the calculations:

- Cases and wastewater detections from Luggage Point upstream I wastewater catchment (Figure 1) and cases from the SA2s corresponding to the Luggage Point upstream I wastewater catchment for Brisbane Inner West from 1 July to 30 August 2021;
- Cases and wastewater detections from Cairns North, Cairns South, and Marlin Coast WWTPs (Figure 2) for the Cairns cluster from 22 February to 19 March 2021.

## 3. Results

### 3.1. Case Studies

#### 3.1.1. Brisbane Inner West Cluster, July–August 2021

**Figure 3:**
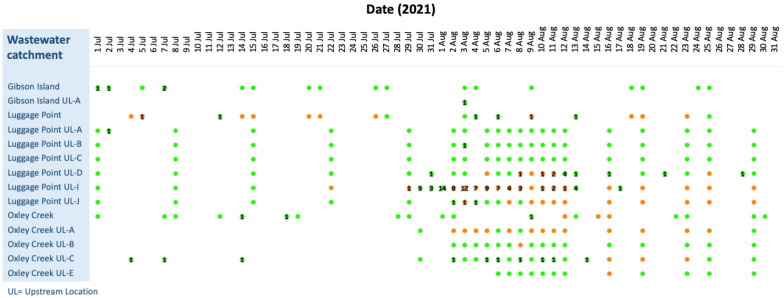
Brisbane Inner West cluster. Daily wastewater detections of SARS-CoV-2 and notified case numbers of COVID-19 across the Luggage Point, Oxley Creek, and Gibson Island wastewater treatment plants and upstream sampling sites from 1 July to 30 August 2021. Orange = positive wastewater detections, green = wastewater samples collected but SARS-CoV-2 not detected. Numbers = reported cases in each catchment.

**Figure A1:**
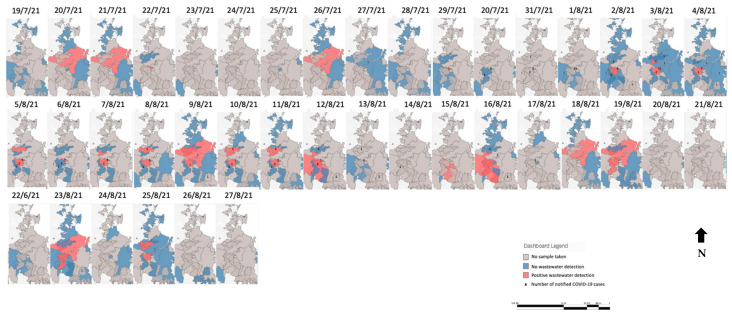
Brisbane Inner West cluster. Maps of positive wastewater detections of SARS-CoV-2 and positive COVID-19 case notifications—Brisbane, Queensland, 15 July–27 August 2021. The darker coloured red represents positive samples from upstream catchments when samples from the whole sewerage treatment plant catchment are also positive for SARS-CoV-2.

Context:

One of the first and largest documented clusters of COVID-19 in Brisbane in 2021 was the ‘Brisbane Inner West cluster’ that occurred between the end of July and end of August 2021 (Figure A1). The cluster included children from two schools and spread to other parts of Brisbane, resulting in 98 reported cases. All positive cases at that time were isolated in hospital after identification. Figure 3 shows the breakdown of each wastewater sample detection/non-detection over time, coupled with the number of cases notified on each day.

The early warning:

On 22 July 2021, an upstream wastewater sample from the Luggage Point I catchment (Figure 3), which services parts of the St Lucia, Indooroopilly, Toowong, and Taringa suburban areas (Figure 1), tested positive for SARS-CoV-2 RNA. As there were no confirmed COVID-19 cases in the Luggage Point upstream I catchment or the surrounding upstream catchments in the previous nine days, the detection was noteworthy. A single positive COVID-19 case was reported in Oxley Creek upstream C on 14 July 2021; however, due to its distance from Luggage Point upstream I, the case was thought unlikely to have been linked to the wastewater detection.

**Figure 4:**
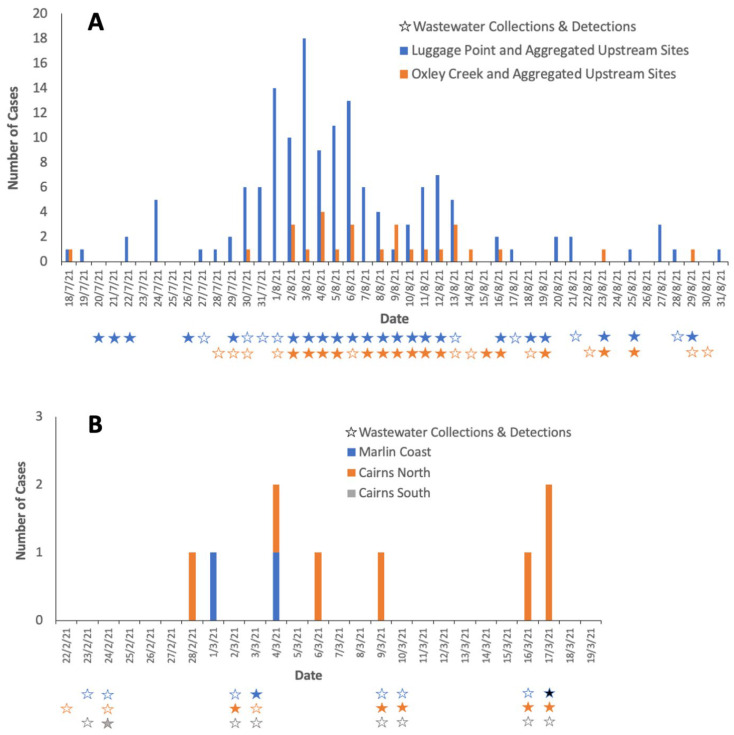
(**A**) Brisbane Inner West cluster. COVID-19 case notifications per statistical area 2 region and wastewater detections in related catchments from 15 July to 31 August 2021; (**B**) Cairns cluster. COVID-19 cases and wastewater detections from three wastewater treatment plant catchments in from 22 February to 19 March 2021. Cases per day are shown in bars. For each catchment, hollow stars indicate no wastewater detections, solid coloured stars indicate positive detections, and absence of a star indicate that no samples were collected.

Day 1: The first case:

On 29 July 2021, the first positive COVID-19 case was reported in Taringa, in the Luggage Point I catchment. Widespread contact tracing and testing of people across Brisbane over the following weeks detected 98 cases of COVID-19 between 29 July 2021 and 17 August 2021. (Figure 4A) Wastewater samples for Luggage Point upstream I catchment returned a positive result on 29 July.

Week 1:

While no wastewater samples were collected in the Luggage Point upstream I catchment from 30 July to 1 August (Figure 3), 5, 3, and 14 cases of COVID-19 residing in the catchment were notified on the 30 and 31 July, and 1 August, respectively. In the adjoining wastewater catchments, samples from both Oxley Creek upstream A and upstream C, which are south of Luggage Point upstream I (Figure 1), were negative on 30 July, despite a positive case being reported on that day in the Oxley Creek upstream C catchment. On 31 July, a positive case of COVID-19 was notified from the Luggage Point upstream D catchment and two cases were notified from the Oxley Creek upstream A catchment. Samples from Luggage Point upstream I and Oxley Creek upstream A were positive throughout week 1, except for Oxley Creek upstream A on 6 August 2021. On 3 August 2021, Luggage Point upstream I and Oxley Creek upstream A both returned positive samples, and had 12 and 1 notified cases of COVID-19, respectively. Additionally, Luggage Point upstream J also reported a notified case of COVID-19 and returned a positive wastewater sample (Figure 4A).

Weeks 2 and 3:

From 3 to 12 of August 2021, wastewater samples from Luggage Point upstream I tested positive and there were between 1 and 12 cases of COVID-19 per day among people residing in the catchment (except on 9 August 2021, when no cases were reported). Samples from Oxley Creek upstream A were positive from 2 to 12 August, with no notified cases in the catchment. There were a few notified cases that resided in the Oxley Creek upstream C catchment in this period, but no wastewater samples from this catchment tested positive during that time (Figure 3).

End of the outbreak:

Positive signals continued to be detected in Luggage Point upstream I and upstream J wastewater samples until 29 August; and in Oxley Creek upstream C until 23 August, and in upstream A wastewater samples until 25 August 2021. The last positive case detected as part of this cluster was on the 17 of August 2021 in the Luggage Point upstream I catchment in Taringa (Figure 3).

#### 3.1.2. Cairns Cluster, February–March 2021

**Figure A2:**
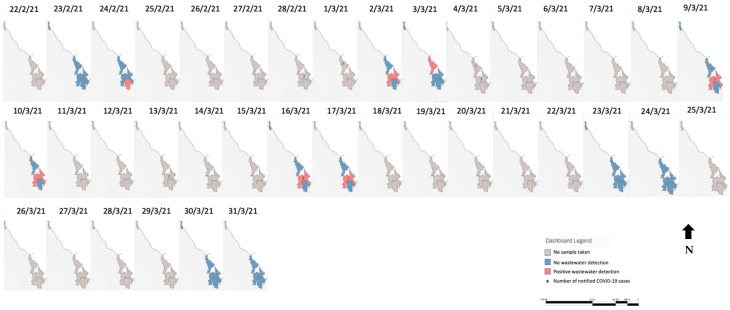
Cairns cluster. Maps of positive wastewater detections of SARS-CoV-2 and positive COVID-19 case notifications—Cairns, Queensland, 22 February–30 March 2021.

Context:

Cairns is a regional city and popular tourist destination in Far North Queensland. Prior to the small outbreak in late February 2021 (Figure A2), only 11 cases, primarily in returned travellers, were notified between May 2020 and 28 February 2021. Cases were isolated in hotels or hospitals.

The Early Warning:

SARS-CoV-2 RNA was detected in a WWTP sample taken from the Cairns South catchment (Figure 2) on 24 February 2021, and a COVID-19 case residing in the Cairns North catchment area was notified on 28 February, with a positive case residing in the Marlin Coast catchment area being notified on 1 March. No wastewater samples were taken from the 25 February until 1 March from any WWTPs in Cairns.

Weeks 1–3:

Samples collected from the Cairns North and Marlin Coast WWTPs on the 2 and 3 March 2021 tested positive. On 4 March, there was one notified COVID-19 case residing in each catchment (Figure 4B). No wastewater samples were collected from 5 to 8 March. Samples from the Cairns North WWTP on 9 and 10 March tested positive, and there was a notification of a case of COVID-19 in a resident of the catchment on 9 March. No wastewater samples were collected from 11 to 15 March. Samples collected from the Cairns North WWTP on 16 and 17 March tested positive, and there were three notified cases of COVID-19 across the two days in residents of this catchment. No wastewater samples were taken from 18 to 22 March, and samples from all three WWTP collected on 23 and 24 March 2021 were negative.

### 3.2. Positive and Negative Predictive Value of Wastewater Detection of Viral RNA for Notifications of Human COVID-19 Cases in Low-Transmission Settings

The PPV of wastewater samples from Luggage Point upstream I for notified cases within this catchment during the period 1 July to 30 August 2021 was 71% and NPV was 95%. The PPV of wastewater samples from all three WWTPs in the Cairns region for predicting notified cases residing within the region was 50% and the NPV was 100% (Table 1).

## 4. Discussion

Our results demonstrate that in settings with low or near-zero COVID-19 transmission, wastewater surveillance can provide an early warning about community transmission, particularly when utilising upstream samples. Our study finds that SARS-CoV-2 RNA detections in wastewater provide an early warning of COVID-19 cases in the community. We also find that testing of wastewater samples has high PPVs (71% in Brisbane Inner West and 50% in Cairns), in spite of a low prevalence. We also find high NPVs (94.7% and 100% respectively), which is not surprising in a low-transmission setting. Positive wastewater detections continued after the date of notification of the last confirmed case in both clusters. This could be a result of continued viral shedding from the notified COVID-19 cases [20], and/or the presence of undetected mild or asymptomatic cases in the catchment [21].

In the Brisbane Inner West cluster, SARS-CoV-2 RNA was not detected in wastewater samples from the Luggage Point WWTP even though cases were occurring in the catchment. This is not unexpected given the large catchment size of Luggage Point, resulting in the dilution of RNA in the wastewater [15]. Upstream sampling involving only a portion of the catchment provided useful information that better reflected COVID-19 cases in a smaller area.

While viral RNA fragments can be detected in wastewater, many factors may influence the usefulness and interpretation of the results. Evidence suggests that while coronaviruses do not remain viable in wastewater, viral RNA fragments remain detectable [22,23]. Dilution of samples may be a limitation in WWTP catchments with large populations when the incidence of human cases is low. Results can also be influenced by frequency and number of collections, variable sensitivity and specificity between upstream and downstream sampling sites, and dilution of virus fragments after rainfall events [24,25,26]. Having ready access to timely availability of data about notification and location of confirmed cases enables better interpretation of the results.

A key limitation of the usefulness of wastewater detections as an early warning system for human COVID-19 cases in Queensland was infrequent wastewater sampling. At many sites, samples representing one 24 h period were only taken once or twice a week due to resource limitations, except during the Brisbane Inner West cluster, where daily samples were taken when the cluster was first detected. Infrequent sampling reduces the potential of wastewater testing to be an early warning system, because detections from incubating or asymptomatic cases can be missed between samples. Increased frequency of sampling using autosamplers would require additional resourcing by local utilities, who were not remunerated for their sampling. This is unlikely to be practical or acceptable to many utilities, as it is not their core business. Increasing the frequency of wastewater sampling also incurs more laboratory costs. At the time of our study, there was no consensus among public health authorities in Queensland about the usefulness of wastewater sampling for early detection of COVID-19 outbreaks, and, therefore, there was an unwillingness for further resourcing of the program. An early warning of COVID-19 transmission in the communities of the two case studies may have triggered measures such as increased testing, reducing the number of subsequent cases and possibly avoiding a costly lockdown. However, it is impossible to know whether these measures would have contained the outbreak. Despite these limitations, our case studies demonstrate the potential value of using wastewater surveillance for early warning in low-transmission settings.

We created web-based dashboards to facilitate visualisation of both wastewater and human data over time and place, and to provide public health decision-makers with easy access to the data. However, due to restrictions and delays in accessing data, COVID-19 human case data were only included in the dashboards from November 2021. Shortly afterwards, Queensland began to experience a surge in community cases due to the emergence of the Omicron variant and the subsequent relaxation of public health measures, including the reopening of interstate and international borders in late December 2021. Consequently, the early warning functionality of wastewater surveillance was no longer useful due to the large number of community cases across the state. While not being useful as an early warning system, the dashboard may have provided unique insights into patterns of wastewater detection as outbreaks and clusters evolved. Such data visualisation techniques are valuable for making complex data and information more accessible and usable for public health decision-makers. While there are large numbers of COVID-19 cases in Australia and elsewhere, early warning systems may not be useful, but our findings suggest that wastewater surveillance may be valuable as an early warning system for future emerging infectious diseases [21,27,28].

## 5. Conclusions

Our two case studies demonstrate that in low COVID-19 transmission settings, wastewater surveillance can function as an early warning tool. Our findings also demonstrate the usefulness of upstream sampling in larger population catchments to better identify the source of clusters and outbreaks. Reliable and representative wastewater sampling methods and timeliness of access to case data are critical for optimising the utility of wastewater surveillance.

## Figures and Tables

**Table 1:** Positive predictive value (PPV) and negative predictive value (NPV) of wastewater detections for the presence or absence of notified COVID-19 cases per catchment cluster in Luggage Point upstream I (1 July 2021–30 August 2021) and Cairns (22 February–19 March 2021).

Cluster	Sampler	Total Number of Notified COVID-19 Cases during Cluster	Total Number of Wastewater Samples Collected	Number (%) of Positive Wastewater Samples	PPV (95% CI)	NPV (95% CI)
Brisbane Inner West (Luggage Point upstream I)	Passive	98	46	21 (45.7%)	71.4% (58.8, 99.1)	94.7% (84.5, 100)
Cairns	Active	9	23	7 (30.4%)	50.0% (5.3, 94.7)	100% (0, 100)

## Data Availability

Data could be made available upon request.
